# Poisson’s Ratio of Closed-Cell Aluminium Foams

**DOI:** 10.3390/ma11101904

**Published:** 2018-10-07

**Authors:** Jaroslav Kováčik, Liviu Marsavina, Emanoil Linul

**Affiliations:** 1 Institute of Materials and Machine Mechanics, Slovak Academy of Sciences, Dúbravskácesta 9, 845 13 Bratislava, Slovak Republic; 2 Department of Mechanics and Strength of Materials, Politehnica University of Timisoara, 1 Mihai Viteazu Avenue, 300 222 Timisoara, Romania; liviu.marsavina@upt.ro (L.M.); emanoil.linul@upt.ro (E.L.)

**Keywords:** aluminium foam, closed-cell foam, Poisson’s ratio, porosity, nondestructive testing, modulus of elasticity

## Abstract

A nondestructive impulse excitation technique was used to investigate Poisson’s ratio of powder metallurgical pure closed-cell aluminium foams according to ASTM E 1876 within the foam density range of 0.430–1.390 g·cm^−3^. Instead of a constant value of 0.34, as according to Gibson and Ashby’s assumption for the Poisson’s ratio of metallic foams, the decrease of the Poisson’s ratio with decreasing foam density was observed. Observed Poisson’s ratio data were in the range of 0.21–0.34. To check the validity of the results, the Young’s modulus was calculated using Poisson’s ratio and its dependence on relative density was successfully modelled using the usual power law function with characteristic exponent of 1.72 ± 0.1. This confirms that the obtained experimental results for Poisson’s ratio are valid. Finally, rule of mixture and percolation theory were used to model the observed decrease of Poisson’s ratio with increasing porosity.

## 1. Introduction

When a material is compressed in one direction, it usually tends to expand in the other two directions perpendicular to the direction of the compression. This phenomenon is called the Poisson effect. Poisson’s ratio *ν* is a measure of this effect. Conversely, if the material is stretched rather than compressed, it usually tends to contract in the directions transverse to the direction of stretching. Again, the Poisson’s ratio will be the ratio of relative contraction to relative expansion and will have the same value as above. The Poisson’s ratio of a stable, isotropic, and linear elastic material cannot be less than −1.0 not greater than 0.5 due to the requirement that Young’s modulus, the shear modulus, and bulk modulus have positive values. Most materials have Poisson’s ratio values ranging between 0 and 0.5. In certain rare cases, a material (called auxetic) will actually shrink in the transverse direction when compressed (or expand when stretched), which will yield a negative value of the Poisson’s ratio [1].

Aluminium foams, due to their metallic character and lightweight properties, have attracted interest from the science and technology communities since 1990 [2,3]. Obviously, their origin goes back to the beginning of the 20th century [4], and they were periodically reinvented and forgotten until 1990 [5,6,7,8,9,10]. Many articles, books, and conference proceedings have been published since 1990 in this field, mostly focusing on mechanical properties [11,12,13,14,15,16,17], and some of them also on thermal and electrical conductivities [18,19]. There is only one exception: there is no published paper regarding the experimental measurement of Poisson’s ratio dependence on density.

There are several reasons for this. Basically, in industrial applications where foams are used (usually, as impact energy absorbers [12]), this value is not so important. Therefore, for FEM (Finite Element Method) calculations, the Poisson’s ratio is often considered to be equal to the bulk aluminium Poisson’s ratio. On the other hand, in the case of aluminium foams, it is not a simple task to measure this value experimentally due to the cellular structure. Moreover, thanks to early plastic/brittle deformations of foams, even at small loads for such highly porous materials, it may also be that the continuum assumption is not reasonable.

It is generally assumed (according to Gibson and Ashby [20]) that the Poisson’s ratio of aluminium foams is independent on porosity and the value is equal to the Poisson’s ratio of used aluminium alloy 0.31–0.34 [20,21]. On the other hand, for modelling purposes in the plastic region, it is usually assumed that plastic Poisson’s ratio of aluminium foams is zero [20]. This situation is usually found also outside aluminium foams. Scientists from many different fields in the finite-element simulation of porous materials or ceramic during FEM calculations in the elastic or ductile regimes usually assume that *ν* is fixed at ~0.3. [22]. However, the latest findings in the research of nanoporous gold are contradict this: Poisson’s ratio values of nanoporous gold around 0.18 have been observed. These are significantly lower than Poisson’s ratio of pure gold (0.42) [23]. The experimental results in this field also indicate that the plastic Poisson’s ratio of the investigated nanoporous gold is nonzero. Therefore, it is another reason to experimentally measure Poisson’s ratio of aluminium foams.

Due to effects of clamping and plastic deformation of very thin cell walls at low stress levels, it is not easy to obtain the elastic properties of aluminium foam from tensile/compressive tests. Usually, it is done by performing a loading–unloading cycle during testing; however, there is already a certain level of plastic deformation even at early stages of stress that affects the results. Instead of tensile/compression tests or indentation tests, it is more appropriate to use nondestructive testing methods. One possibility is to use free vibrations of specimens of a given geometry. This vibration technique has been used for the determination of Young’s modulus of aluminium foams since 1990s [3]. However, this requires long rods with a length much larger than the diameter of the rod. In this case, the modulus is proportional to the density and squared resonant frequency. Sadly, the determination of shear modulus is very sensitive to the heterogeneity inside the foam. Therefore, it is not possible to use this technique for Poisson’s ratio measurement. The impulse excitation technique (IET) [24] is a nondestructive material characterization technique used to find the elastic properties and internal friction of a material of interest. It measures the resonant frequencies to calculate the Young’s modulus, shear modulus, Poisson’s ratio, and internal friction of disc-shaped samples. The measurement principle is based on tapping the sample with a small projectile and recording the induced vibration signal with a microphone or laser vibrometer. Afterwards, the acquired vibration signal in the time domain is converted to the frequency domain by a fast Fourier transformation. Dedicated software can determine the resonant frequency with high accuracy to calculate the elastic properties based on the classical beam theory. The advantage of this method is that it is applicable to porous and brittle materials due to small strains. Therefore, it was expected that the new vibration techniques would enable us to measure Poisson’s ratio of aluminium foams directly. The aim of the present work was to use the IET to measure the Poisson’s ratio of pure aluminium foam and to confirm its independence on porosity.

## 2. Experimental

**Figure 1:**
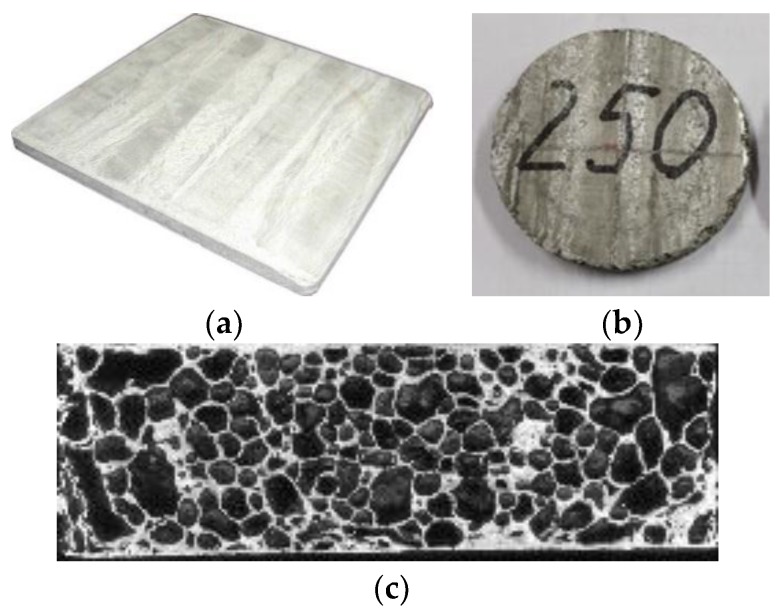
(**a**) Prepared aluminium foam plate, (**b**) disc sample for the impulse excitation technique (IET)test, and (**c**) typical cell structure of foam, density 0.50 g·cm^−3^.

Aluminium foam samples were prepared via a powder metallurgical route from aluminium alloy powder Al 99.7 (Fe 0.13 wt. %, Si 0.10 wt. %, Al balanced, Mepura GmbH (Ranshofen, Austria), powder size 63–400 µm) that was mixed together with a foaming agent (0.7 wt. % of TiH_2_, Chemetall GmbH, Frankfurt am Main, Germany, Grade N, grain size below 60 µm, powder size d_50_ = 14.52 µm) cold isostatic pressed and then continuously hot extruded at 500 °C into a foamable precursor. The various quantities of precursor were foamed up in a steel mold at 800 °C for different times to obtain different foam densities. The prepared foams were flat plates with the dimensions of 140 × 140 × 8.3/9.61 mm^3^ (Figure 1a). The foamed plates’ densities were in the range of 0.430–1.550 g·cm^−3^. Particularly, foams with higher densities were prepared by interrupting the foaming process at early stages of gas evolution from the foaming agent and were more anisotropic as low-density foams.

The samples for the determination of Poisson’s ratio of aluminium foams were cut using a milling machine from foam plates. The typical geometry of the samples was around disc of *φ* 60 mm × 8.3/9.61 mm (see Figure 1b). A foam disc with a density of 1.550 g·cm^−3^ was not suitable for the determination of its Poisson’s ratio as its resonant frequency ratio *f*_1_/*f*_2_ (see below for definition) was outside of the range required by the used measurement method. However, this result indicated that the used measurement method was precise enough to detect heterogeneous and anisotropic samples. Therefore, only the density range of 0.430–1.390 g·cm^−3^ for round discs was further used for the results and discussion.

**Figure 2:**
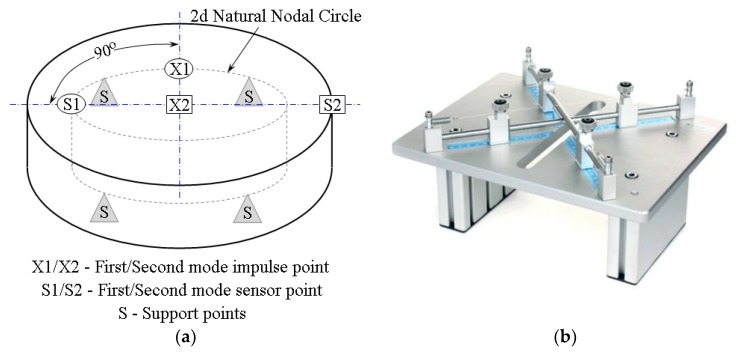
(**a**) Support, impulse, and sensor points for first and second natural vibrations in discs according to ASTM E 1876. (**b**)Picture of used precision wire support for foam disc samples.

An IET was used to measure Poisson’s ratio using a Resonant Frequency & Damping Analyser (IMCE, Genk, Belgium). To determine the Poisson’s ratio of aluminium foams, vibrational tests were carried out on round disc samples according to ASTM E 1876 [24]. From the ratio between the flexural and the antiflexural frequency (see Figure 2), it is possible to calculate the Poisson’s ratio using a numerical solution by Martincek [25] and Glandus [26].

Basically, the Poisson’s ratio is determined using the resonant frequencies of the first two natural vibration modes. The dynamic Young’s modulus and dynamic shear modulus are then calculated using the Poisson’s ratio, the experimentally determined fundamental resonant frequencies, and the specimen dimensions and mass. The first natural vibration occurs when the displacements in the cross-sectional plane (the plane that is parallel to the flat of the disc) are normal to the plane and symmetrical around two orthogonal diameters in the plane of the disc, producing a twisting of the disc. This is an orthogonal antiflexural mode of vibration. For the first natural vibration mode, the nodes are located along two orthogonal diameters, offset 45° from the point where the vibration was induced. The antinodes are located along two orthogonal (90° offset) diameters in the disc, with one diameter intersecting the point where the vibration was induced.

The second natural vibration occurs when the displacements in the cross-sectional plane (the plane that is parallel to the flat of the disc) are normal to the plane and are uniform in displacement for a given radial distance from the centre point through the entire 360° arc. This is axisymmetric flexural vibration. For the second natural vibration mode of a disc, the nodes are located in a circle concentric with the centre of the disc with a fractional radius of 0.681 of the disc radius. The antinodes are located at the centre and around the circumference of the disc specimen.

The derivation and use of the equations for calculating the Poisson’s ratio and moduli from disc-shaped specimens are described in detail in References [25,26]. Martincek [25] gives the derivation and procedures for the baseline calculation. The fundamental equation defining the relationship between the natural resonant frequency, the material properties, and the specimen dimensions is given by Martincek as:

(1) $$f_{i}=\frac{K_{i}}{2\pi r^{2}}\sqrt{\frac{A}{\rho t}}$$

where *f*_i_ is the resonant frequency of interest, *K*_i_ is the geometric factor for that resonant frequency, *r* is the radius of the disc, *A* is the plate constant (*A* = *Et*^3^/[12 × (1 − *ν*^2^)], *t* is the disc thickness, *ρ* is the density of the disc, *E* is Young’s modulus of elasticity, and *ν* is the Poisson’s ratio for the disc material. This is a general equation that is valid for both the first natural and second natural vibrations. Glandus [26] supplements the Martincek article with more extensive tables for the geometric factors *K*_i_ and for determining Poisson’s ratio. The Poisson’s ratio is determined directly from the experimental values for the first and second natural resonant frequencies given in tables in the above mentioned references [25,26] and as well in ASTM E 1876 [24]. There, the value for Poisson’s ratio (*ν*) is interpolated from the table using the ratio of the second natural resonant frequency to the first natural resonant frequency (*f*_2_/*f*_1_) correlated with the ratio of the specimen thickness to the specimen radius (*t*/*r*). The following requirements must be met: the ratio of the flexural and antiflexural resonant frequencies is in the range 1.35 ≤ *f*_1_/*f*_2_ ≤ 1.90, and diameter *D* and thickness *t* of the specimen ought to be larger than the ratio *D*/*t* ≥ 4.

For the Young’s modulus of a disc, two calculations of *E* (*E*_1_ and *E*_2_) are made independently from the two resonant frequency measurements, and then a final value *E* is determined by averaging the two calculated values *E*_1_ and *E*_2_:

(2) $$E_{1}=\left[12\pi f_{1}^{2}D^{2}m\left(1-\nu^{2}\right)\right]/\left(K_{1}^{2}t^{3}\right)$$

(3) $$E_{2}=\left[12\pi f_{2}^{2}D^{2}m\left(1-\nu^{2}\right)\right]/\left(K_{2}^{2}t^{3}\right)$$

where *E*, *E*_1_, and *E*_2_ is the average, first, and second calculation of Young’s modulus, respectively. There, *f*_1_ is the first and *f*_2_ is the second natural resonant frequency of the disc, *D* is the diameter and *m* is the mass of the disc, *ν* is Poisson’s ratio for the specimen as determined previously, *K*_1_ is the first natural geometric factor from the table (function of *t*/*r* and *ν*) and *K*_2_ is the second natural geometric factor from the other table (again, function of *t*/*r* and *ν*), *t* is the thickness of the disc, and *r* is the radius of the disc.

To properly determinate the elastic properties of the disc specimens, it is necessary to fulfil conditions of the specimen geometry and the proper positioning of the support points below the sample and the impulse and sensor points above the sample (see Figure 2) [27]. According to ASTM, the ratio between the diameter and the thickness of the specimen must be at least 4. For all investigated foam samples, this ratio was found to be in the range of 6–8. Then, a support nodal circle diameter was calculated as 0.681 for the specimen diameter of each disc, leading to the value of 40.86 mm. Finally, the sample was freely excited by a light impact strike with a special hammer at impulse points (X1 and X2 in Figure 2) and the resonant frequency was measured by a microphone placed in the sensor point for both the first and second excitation modes (S1 and S2 in Figure 2). To ensure repeatability, at least five readings of resonant frequency were done and the average value was used for the first and the second natural frequencies, respectively. Measured resonant frequencies for all samples had readings with a variation smaller than 1%, which agrees with ASTM E 1876. Finally, all flexural and antiflexural resonant frequencies were in the range of 1.35 ≤ *f*_1_/*f*_2_ ≤ 1.90 for all samples, except the abovementioned one for the densest sample. Finally, Poisson’s ratio was determined directly from the measured resonant frequencies using the tabulated values of *f*_1_/*f*_2_ and *t*/*r*. Then, the Young’s modulus values were determined via Equations (2) and (3) using the corresponding ASTM tables.

## 3. Results and Discussion

The Poisson’s ratio of pure aluminium and aluminium alloys is in the range of 0.31–0.34 [20]. In Gibson and Ashby’s book, they put forward the opinion that the Poisson’s ratio of aluminium foams is equal to the Poisson’s ratio of the used matrix metal or metal alloy and is independent of density [21]. To avoid potential reactions and consequent phase creation inside of the foam cell walls, pure aluminium powder was used in this study for the foam preparation. Therefore, the foam cell walls’ composition is quite simple. There are present aluminium, Ti localized inside of cell walls (0.7 wt. % from decomposed foaming agent),and finally, alumina (around 1 wt. % from powder envelopes, unfortunately not visible on SEM microscope) [11]. This means that there are no stabilizing additions such as 10–20 vol.% of Al_2_O_3_, SiC, TiB_2_, etc. Therefore, it can be expected that the Poisson’s ratio value of 0.34 will be observed within all the investigated porosity ranges.

**Figure 3:**
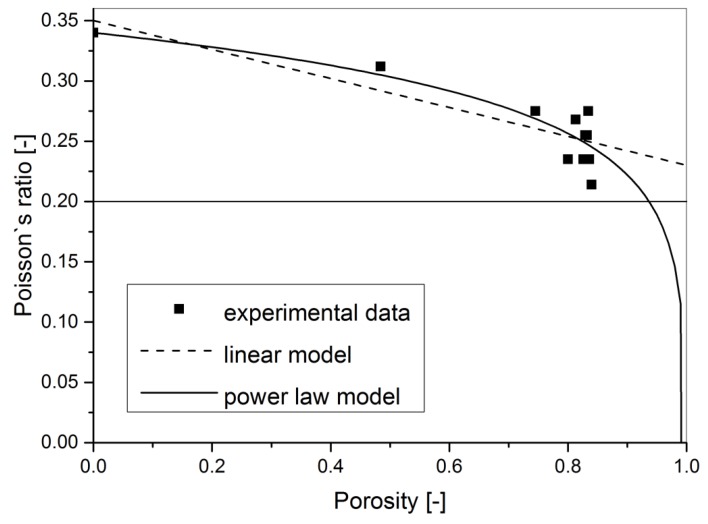
The porosity dependence of Poisson’s ratio of pure aluminium foams. Plotted are the linear model, the power law model, and also the 0.2 constant.

Unfortunately, the Poisson’s ratio of the pure aluminium foam measured using the impulse excitation nondestructive technique in the density range of 0.430–1.390 g·cm^−3^ showed a different result. The Poisson’s ratio of closed-cell pure powder metallurgical PM aluminium foam is density dependent (see Table 1 and Figure 3). It was experimentally observed that with decreasing density, the Poisson’s ratio of aluminium foams decreases from the value of 0.34 down to 0.21. This result is similar to the porosity dependence of Poisson’s ratio observed previously in porous solids and ceramics [28,29].

The Young’s modulus data according to Equations (2) and (3) were calculated to check the validity of the obtained Poisson’s ratio results. Further, the Young’s modulus dependence on porosity modelled using percolation [18] or Gibson and Ashby’s power law model [20] was

(4) $$E=E_{0}{\left(1-\phi \right)}^{f_{E}}$$

where *E*, *E*_0_ is foam modulus and modulus of bulk aluminium, *φ* is the porosity, and *f*_E_ is the characteristic exponent for the power law dependence of Young’s modulus. It is usually accepted that characteristic exponent of aluminium foams is in the range of 1.8–2.2 [20].

**Figure 4:**
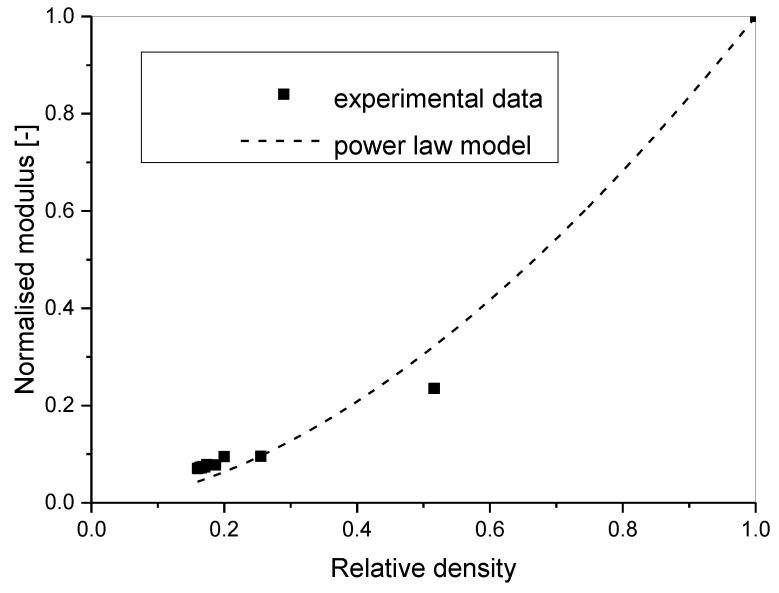
The dependence of the normalised Young’s modulus of pure aluminium foams on relative density. Plotted line is the power law model with *R*^2^ = 0.982, *f*_E_ = 1.72 ± 0.10.

The fitting results for the density dependence of Young’s modulus led to the characteristic exponent of *f*_E_ = 1.72 ± 0.10 with *R*^2^ = 0.982 (Figure 4). The observed value mostly coincided with the experimental results for various types of aluminium foams between 1.8 and 2.2 [20]. The lower value of the characteristic exponent is probably due to the certain anisotropy of the foam samples.

It can be concluded that the obtained Young’s modulus experimental results and the validity of the power law model for Young’s modulus porosity dependence proved that the observed Poisson’s ratio decrease with increasing porosity was correctly measured.

Therefore, the originally accepted hypothesis that the Poisson’s ratio of aluminium foams is constant at all density ranges and has a value of 0.34 was proved to be incorrect in the case of closed-cell pure aluminium PM-prepared foams. It implies that the hypothesis can be also incorrect for other types of aluminium and metallic foams.

The elastic Poisson’s ratio of open-cell AlSi0.5Mg aluminium foams was investigated numerically by Wicklein and Thoma [30]. Finite-element discretization, which has been derived from real foam specimens by computer tomography data, was used. They found that Poisson’s ratio is approximately constant, with an average value of 0.23 in the relative density range of 0.35–0.5. The Poisson’s ratio value for open-cell AlSi0.5Mg foams is smaller than the Poisson’s ratio value for the AlSi0.5Mg alloy, thus supporting the experimentally observed dependence of Poison’s ratio on porosity in the present paper.

Due to the chosen matrix composition (pure Al with 0.7 wt. % Ti), we can exclude potential phase creations inside foam cell walls affecting the mechanical properties of the investigated foams. For this reason, only the porosity and heterogeneity of the foam structure can affect the Poisson’s ratio values of the investigated foams. From Figure 1c, it is clear that some scatter of experimental data for low-density foams (with 80–84% porosity) took place. The reason for this can be the increase of pore size with decreasing density.

It is usually expected for metallic foams that the geometry of the sample contains in all directions at least 10 pores to avoid certain heterogeneity effects. In our case, the average pore size is around 0.8–1 mm at the density of 0.50 g·cm^−3^. This means that the local variations of porosity affect the measured values for 80–84% porosity, thus leading to the large scatter of experimental data. With increasing density, the pore size decreases, thus diminishing this effect [11].

When the pure aluminium Poisson’s ratio of 0.34 was used in the data-set, there were three points outside the clustered data points for 80–84% porosity. Even when only data for 0%, 48%, and 75% porosity were used, the observed decrease of Poisson’s ratio was obvious due to the higher homogeneity of the foam structure for these points.

It can be concluded that the higher the foam porosity, the higher the scatter of the measured data. This is due to the fact that all IET methods based on vibrations need 2D samples due to the beam theory calculation with the third dimension being as small as possible. Therefore, certain error will always be there in the case of metallic foams.

In principle, it is possible to calculate the porosity dependence of Poisson’s ratio from two elastic moduli, e.g., shear (*G*) and longitudinal Young moduli (*E*):

(5) $$\nu =\frac{E}{2G}-1$$

While there are many models that can predict the porosity dependence of elastic and shear modulus, models to predict Poisson’s ratio *ν* are mostly developed from Equation (5), and many of them are very complex. As the observed experimental results for Poisson’s ratio had high data scatter, the focus was on models and explanations that were as simple as possible.

Most models created for porous solids which are based on Equation(5) lead to the conclusion that with increasing porosity, there is a tendency for the Poisson’s ratio of porous ceramic to approach the constant value [28,29] of 0.2, as this value seems to be an “attractor“ for the effective Poisson’s ratio. However, when the Poisson’s ratio of the solid phase *ν*_0_ is below 0.2 (*ν*_0_ < 0.2), the effective Poisson’s ratio tends to increase with increasing porosity to this value.

A similar attracting behaviour was proposed originally by Kitazono et al. [31] for metallic foams using a continuum micromechanical model (with the equivalent inclusion method and the mean-field approximation). They stated that Poisson’s ratio of foam decreases with the decrease of the relative density if Poisson’s ratio of matrix metal >0.2 and increases if Poisson’s ratio of matrix metal <0.2 in the certain parameter limit (*p* = 0). Only when Poisson’s ratio of matrix metal =0.2 does Poisson’s ratio of foam become independent of the relative density. Since the Poisson’s ratio of most metals and alloys is more than 0.2, the micromechanical model predicts that the Poisson’s ratio of metal foams monotonously decreases with the decreasing of the relative density and approaches a constant value of 0.25 [31]. Observed values in the present paper at low porosity show that the Poisson’s ratio of the investigated foams deviates from this attractor value of 0.25, as lower values of Poisson’s ratio down to 0.21 were measured.

In another approach, the Poisson’s ratio can be in the simplest way modelled by a linear approximation [29]:

(6) $$\nu =\nu_{0}+b\times \phi =\nu_{0}+\frac{3\left(1-5\nu_{0}\right)\left(1-\nu_{0}^{2}\right)}{2\left(7-5\nu_{0}\right)}\times \phi$$

where *ν* is the effective Poisson’s ratio, *ν*_0_ is the Poisson’s ratio of the corresponding bulk material, and *φ* is the porosity. However, this approach has the typical shortcomings of mixture-based theories. In the case of porous ceramics (ceramic composites where one phase consists of air-filled voids exhibiting zero Poisson’s ratio), the rule of mixture for the effective Poisson’s ratio evidently fails, since the effective Poisson’s ratio did not approach the zero value (as should be the case for the rule of mixture) [22].

After fitting the observed Poisson’s ratio results to this linear approximation model, it was clear that it described them well (see Figure 3). The only drawback was that the calculated constant *b* for the bulk aluminium Poisson’s ratio value of 0.34 and fitted values of constant *b* = (1 − 5*ν*_0_)·(1 − *ν*_0_^2^)/(2 × (7 − 5*ν*_0_)) were −0.175 and −0.12 ± 0.02 (see Table 2), respectively. This was a difference of about 31% for constant *b*.

Another possibility is to use percolation theory, which works with a threshold idea that is based on the formation of long-range connectivity in random systems. Below the threshold, a giant connected component does not exist, while above it, there is a giant component of the order of the system size [32,33]. For metallic foam, the percolation threshold is the porosity at which the mechanical properties of the porous material become zero (because the foam does not exist anymore as an entity in the order of the system size).

Therefore, the Poisson’s ratio of solids was modelled using the power law dependence on porosity on the basis of the percolation theory [33] by incorporating the percolation threshold into the model [34,35,36,37]. This leads to the well-known percolation power-law relation or differential approximation [34,38]. As was mentioned above, the percolation threshold is the porosity at which the mechanical properties of a porous material become zero. Garboczi et al. [39] showed that for the special case of overlapping ellipsoids, a percolation threshold above 99.9 vol.% can be observed. Based on this assumption, the percolation equation for the investigated aluminium foams followed a simplified form [34]:

(7) $$\nu =\left(\nu_{0}+1\right){\left(1-\phi \right)}^{f_{\nu }}-1$$

where *f*_*ν*_ is characteristic exponent for Poisson’s ratio.

The observed experimental data were then fitted to Equation (7) and the obtained fitting results are also plotted in Figure 3 and are listed in Table 2. The *R*^2^ value for this model is almost the same as the *R*^2^ value of the linear approximation. Basically, both models can be used to predict the observed Poisson’s ratio dependence on porosity within the investigated porosity range. The only difference is that the Poisson’s ratio dependence based on the percolation model tends to the zero value for porosity approaching 100%.

Summarising, the decrease of Poisson’s ratio dependence on porosity was observed experimentally for closed-cell pure PM aluminium foams. Poisson’s ratio values were in the range of 0.21–0.34. Both the linear approximation and percolation models are suitable to describe the observed decrease within the investigated porosity range.

## 4. Conclusions

Direct measurement of the Poisson’s ratio of PM pure aluminium foam was performed in the density range of 0.430–1.390 g·cm^−3^ via using an impulse excitation technique according to ASTM E 1876. The decrease of Poisson’s ratio with decreasing density of aluminium foams was observed in the range of 0.34–0.21. This contradicts Gibson and Ashby’s assumption that the Poisson’s ratio of aluminium foams is constant at all density ranges and has the Poisson’s ratio value of pure aluminium (0.34).

The percolation model or Gibson and Ashby’s model was used to describe the modulus of elasticity dependence on relative density. The results for Young’s modulus confirmed the validity of Poisson’s ratio measurement as the results for the modulus coincide with previous results observed for the modulus of other aluminium foams. The observed characteristic exponent was 1.72 ± 0.1.

The rule of mixture and the derived percolation model were used to explain the observed behaviour of Poisson’s ratio with porosity. It was shown that both the linear approximation and percolation models are suitable to describe the observed decrease. To determine which model is better, it will be necessary to measure higher-porosity foams, which will be the aim of future work.

## Figures and Tables

**Table 1:** Measured Poisson’s ratio and Young’s modulus values of pure PM aluminium foams.

Density (g/cm^3^)	Relative Density	Porosity	Poisson’s Ratio	E
	(-)	(-)	(-)	(GPa)
0.433	0.160	0.840	0.214	4.93 ± 0.12
0.443	0.164	0.836	0.235	5.13 ± 0.07
0.448	0.166	0.834	0.275	5.03 ± 0.22
0.453	0.168	0.832	0.255	5.20 ± 0.07
0.461	0.171	0.829	0.255	5.17 ± 0.05
0.469	0.174	0.826	0.235	5.49 ± 0.12
0.504	0.187	0.813	0.268	5.45 ± 0.18
0.540	0.200	0.800	0.235	6.65 ± 0.03
0.688	0.255	0.745	0.275	6.68 ± 0.18
1.394	0.516	0.484	0.312	16.47 ± 0.18
2.700	1.000	0.000	0.340	70

**Table 2:** Fitting results for the experimental data for the linear model and the power law model with R squared (coefficient of determination).

Model	Equation	*ν*_0_ Model	*ν*_0_ Fit Result	*b* Theoretical	Fit Result	*R*^2^
Linear	$\nu =\nu_{0+}+b\times \phi$	0.34	0.35 ± 0.02	−0.175	*b* = −0.12 ± 0.02	0.698
Power law	$\nu =\left(\nu_{0}+1\right){\left(1-\phi \right)}^{f_{\nu }}-1$	0.34	0.34 ± 0.02	-	*f_ν_* = 0.04 ± 0.01	0.761
